# DNA Damage in *Euonymus japonicus* Leaf Cells Caused by Roadside Pollution in Beijing

**DOI:** 10.3390/ijerph13070742

**Published:** 2016-07-22

**Authors:** Tianxin Li, Minjie Zhang, Ke Gu, Uwizeyimana Herman, John Crittenden, Zhongming Lu

**Affiliations:** 1 School of Civil and Environmental Engineering, University of Science and Technology Beijing, Beijing 100083, China; 15201451547@163.com (M.Z.); cyrllguke@163.com (K.G.); uwiherman05@yahoo.fr (U.H.); 2 School of Civil and Environmental Engineering, Georgia Institute of Technology, Atlanta, GA 30332, USA; john.crittenden@ce.gatech.edu (J.C.); Zhongming.Lu@gatech.edu (Z.L.); 3 Brook Byers Institute for Sustainable Systems, Georgia Institute of Technology, 828 West Peachtree St., Suite 320B, Atlanta, GA 30332, USA

**Keywords:** comet assay, percentage of DNA in the tail, tail moment, air pollution

## Abstract

The inhalable particles from vehicle exhaust can cause DNA damage to exposed organisms. Research on DNA damage is primarily focused on the influence of specific pollutants on certain species or the effect of environmental pollution on human beings. To date, little research has quantitatively studied the relationship between roadside pollution and DNA damage. Based on an investigation of the roadside pollution in Beijing, *Euonymus japonicus* leaves of differing ages grown in heavily-polluted sections were chosen as biomonitors to detect DNA damage using the comet assay technique. The percentage of DNA in the tail and tail moment was chosen as the analysis index based on SPSS data analysis. The roadside samples showed significantly higher levels of DNA damage than non-roadside samples, which increased in older leaves, and the DNA damage to *Euonymus japonicus* leaf cells was positively correlated with haze-aggravated roadside pollution. The correlation between damage and the Air Quality Index (AQI) are 0.921 (one-year-old leaves), 0.894 (two-year-old leaves), and 0.878 (three-year-old leaves). Over time, the connection between DNA damage and AQI weakened, with the sensitivity coefficient for δ_year 1_ being larger than δ_year 2_ and δ_year 3_. These findings support the suitability and sensitivity of the comet assay for surveying plants for an estimation of DNA damage induced by environmental genotoxic agents. This study might be applied as a preliminary quantitative method for Chinese urban air pollution damage assessment caused by environmental stress.

## 1. Introduction

The concentration of inhalable particles, such as nitrogen oxides, hydrocarbons, and carbon monoxide from vehicle exhaust, can be higher along roadways, which can cause DNA damage to exposed organisms [1]. Many diseases caused by mutations in DNA have been recently recognized as related to toxic substances in the air where air pollution is serious. Research on DNA damage is primarily focused on the influence of specific pollutants on certain species or the effect of environmental pollution on human beings, especially on policemen, middle-aged and elderly women [2,3,4,5]. Bartato [6] indicated that oxidative stress is one of the mechanisms through which traffic-related air pollution causes adverse effects on human health; Møller [7] indicated that air pollution particles generate oxidatively-damaged DNA by promoting a milieu of oxidative stress and inflammation. Research on plant DNA damage induced by environmental pollution is relatively less compared with studies on human DNA damage. Early in 2003, Chutchalida [8] compared the DNA damage detected with the plant comet assay in roadside and non-roadside environments, their study applied a preliminary method in roadside pollution assessment; however, they failed to quantify the connection degree between roadside pollution and the plant DNA damage, and they only performed the test from April to October, observing a small difference between samples. In addition, the plants they selected as biomonitors, ginkgo (*Ginkgo biloba*), pohtos (*Epipremnum aureum*), and periwinkle (*Vinca rosea*), are not common roadside greening plants in China. In the present study, *Euonymus japonicus*, a common roadside greening plant in Beijing, is selected as a biomonitor for detecting the plant DNA damage induced by roadside pollution in Beijing. The connection degree between the roadside pollution and the plant genetic damage has been quantitatively researched. Although some studies have focused on the effect of a single pollutant, especially heavy metals, on sensitive plants in China [9], they have just conducted batch experiments in the laboratory, not testing in a real, complex, polluted environment, and of course people do not know what exact damage has been made by the roadside pollution in Beijing. In the present research, plant samples with different ages are all collected from roadside, thus the DNA damage detection results could be more convincing to reflect the real dangers of the traffic pollution environment. Currently, micronucleus technology, enzyme activity detection, single cell electrophoresis, and polymerase chain reaction are used to assess DNA damage. Ojima [10] used comet assay to quantify the degree of photo-induced DNA damage by using radish sprouts exposed to varied light conditions, and found the IN_D_ (defined to express the DNA intactness) value gradually decreased with increasing light intensity (22–430 W·m^−2^) and exposure time (0–6 h), and ultimately fell to 21% at 6 h under a light intensity of 430 W·m^−2^. Hattab [11], using comet assay, detected the DNA damage in *Pisun sativum* root and leaf cells induced by genotoxic agents (cadmium chloride and copper chloride), and found that root cells are more sensitive to the cadmium compared with the copper, and the DNA damage in leaf cells was only significant when plants were treated with the higher cadmium concentrations. These researchers [12] have used comet assay because of the ease of sample preservation, convenient operation, and high sensitivity, low cost, and wide use. Thus, this research adapted the comet assay as the detection method to evaluate the degree of plant DNA damage induced by traffic pollution.

As Beijing traffic and air pollution continue to increase, environmental pollution problems are now receiving more and more attention [13]. *Euonymus japonicus*, a common evergreen road planting species, is chronically exposed to roadside pollution and is the species most obviously affected by roadside pollution [14]. In this article, leaves from *Euonymus japonicus* planted in several sections of Beijing with typical traffic congestion were collected to conduct DNA damage analysis to examine the biological health of the samples and analyze the impact of roadside pollution quantitatively. For comparison, we also collected samples in less polluted areas as a control.

## 2. Experimental Section

### 2.1. Test Samples

**Figure 1:**
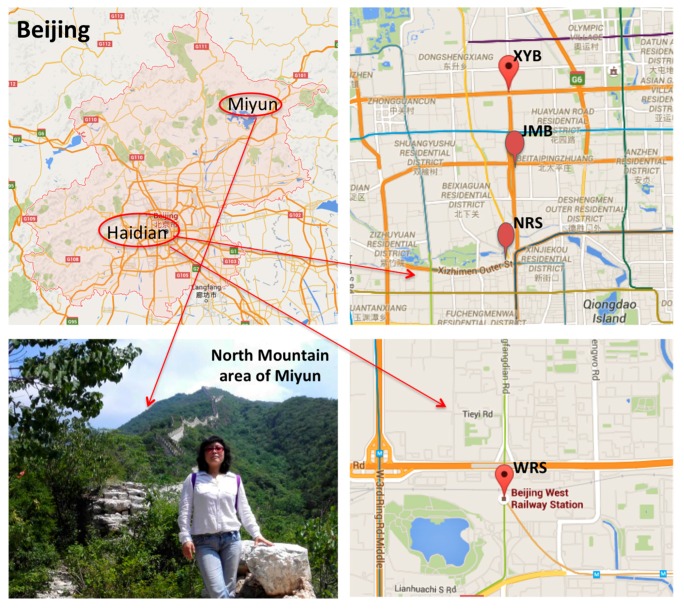
Map of sampling sites: Xueyuan Bridge (XYB), Jimen Bridge (JMB), Beijing Northern Rail Station (NRS), Beijing Western Rail Station (WRS), and the northern mountain area of Miyun.

Sample leaves from *Euonymus japonicus* were collected in five locations in Beijing, with four of them in areas with heavy traffic congestion: Xueyuan Bridge (XYB), Jimen Bridge (JMB), Beijing Northern Rail Station (NRS), and Beijing Western Rail Station (WRS). The last area was located in the northern mountain area of Miyun as a control. We collected three leaf specimens of the same age. At each location, 10 plants were picked randomly, and the leaf specimens of each plant were collected at one, two, and three years old. The one-year-old leaves are new blades, which are tender green and grew intensively in the center of the crown. The two-year-old leaves are dark green on their tops and light green on their bottoms with an obtuse leaf apex and margin serrulate. The three-year-old leaves are dark green on both sides, suborbicular, are smaller in size, and grow sparsely. Three specimens from each age group were collected within a 1 km circumference of these areas and mixed for testing. For each test, 200 randomly chosen nuclei were analyzed. The sampling sites are shown in Figure 1, and the growing conditions of all test species are identical, except for the pollution exposure position.

The concentration of the particulate matter smaller than 2.5 micrometers (PM_2.5_) and the particulate matter smaller than 10 micrometers (PM_10_) were detected from March to May in 2013 (with the exception of rain and snow days) during the sampling period. Samples were taken 2 m from the ground within a circle of two meters (radius) around the sampling points and were detected from 8 a.m to 11 a.m.

PM was measured over this period to assess the air quality, and the leaves were sampled once on 12 March 2013.

### 2.2. Chemicals and Media

Reagents for electrophoresis, normal melting point (NMP) and low melting point (LMP) agarose, and general laboratory reagents were purchased from Biodee Bio Tech Corporation Ltd. (Beijing, China).

Other instruments used include the following: (1) a 8530 dust monitor from TSI Instrument Company (Shoreview, MN, USA); (2) a centrifuge from model number QD16 from AIDA Instrument Company (Shanghai, China); (3) an electronic balance, model number PL203, from Mettler Toledo (Shanghai, China); (4) an electrophoresis system, model number DYY-6C from Liuyi (Beijing, China); and (5) a fluorescence microscope, model number DS-Ri1, from Nikon (Sendai, Japan).

### 2.3. Comet Assay

The comet assay was performed according to the method developed by Singh et al. [15], with a few modifications [16]. After surface sterilization of the collected leaves, small pieces (1 cm^2^) were cut [17]. Ten grams of leaves were weighed and placed in a 60 mm petri dish kept on ice containing 40 mL of grinding medium (20 μmol/L sucrose, 10 μmol/L MgCl_2_, 20 μmol/L Tris-HCl buffer, pH 7.8), and then the samples were ground into a homogeneous suspension using a mortar. The suspension was filtered the using two filters. First, the suspension was passed through a filter (Zhejiang, China) with a nominal pore size of 61 nm, and then the eluate was filtered through another filter with a nominal pore size of 38 nm. The final eluate was centrifuged for 5 min at 200 rpm (R = 10 cm, G = 447.2 g). The centrifuged solids were re-suspended in a buffer solution containing 1 mmol/L MgCl_2_, 0.01 M phosphate-buffered saline（PBS） buffer, and S-buffer (1 mol/L sorbitol and 25 mmol/L phosphate buffer mixed together at pH 6.5) and 1 mmol/L phenyl methane sulfonyl fluoride (PMSF). After centrifugation for 10 min at 2000 rpm (R = 10 cm, G = 447.2 g), sucrose solution was added into the centrifuged precipitate. The sucrose cushion contained 320 mmol/L sucrose in 0.01 M PBS and 1 mmol/L CaCl_2_. Next, the centrifuged solids were re-suspended in S-buffer. The nucleus suspension density was generally 20~40 cells in each field of microscope. When nucleus density is too great, tail overlap would happen in electrophoresis; but too little made it difficult to image. It is recommended that the nuclear isolation be performed in the dark to avoid white light-induced damage [18].

Additionally, because of the manual operation, the cell survival rate was generally between 70% and 95%. Before making the slide, trypan blue stain was used to ensure that the observed cell survival rate was above 85% [19]. The cell suspension was mixed with 0.4% trypan blue solution (diluted with PBS) in 9:1. Under the microscope, the dead cells would be dyed light blue and living cells would be unaffected from staining. Within 3 min, the number of living cells and dead cells were counted, respectively.

**Figure 2:**
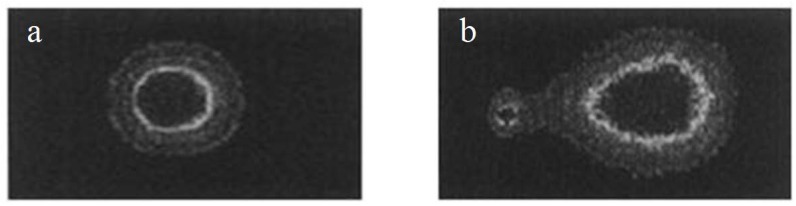
The comet assay images of a healthy cell (**a**) and a damaged cell (**b**). The damaged cell is observed as a bright comet head with a dispersive tail.

The commonly used “sandwich” structure was chosen to perform the comet assay. Regular microscope slides were dipped into a solution of 1% normal melting point (NMP) agarose prepared with 100 μL PBS buffer at 65 °C, and a coverslip was immediately placed on top. After the agarose layer solidified, the coverslip was removed. Then, 20 μL of the cell suspension was mixed with 75 μL of 0.8% LMP (low melting point) agarose and spread over the first layer. The coverslip was replaced on top, and the slide was incubated at 4 °C for 10 min. Then, the coverslip was removed, 80 μL of 1% LMP agarose was added as a third layer, and the cover slip was placed on top again until solidification [20]. Then, the coverslip was removed, and the slide was immersed in freshly prepared ice-cold lysis solution (2.5 mol/L NaCl; 100 mmol/L Na_2_EDTA; 10 mmol/L Tris; pH = 10; 1% sodium sarcosinate; 1% TritonX-100 and 10% DMSO (dimethylsulfoxide） were added immediately before use) for 1 h at 4 °C. Damage to nucleic acids by nucleases can be prevented by the use of cell lysis solution. The solution separates nucleic acids from proteins, thereby stabilizing the nucleic acid structure. Proteins enter the gel and spread into the lysis solution, thereby retaining the nucleic acids. The slide was drained and placed in a horizontal electrophoresis tank filled with freshly prepared alkaline buffer (300 mmol/L NaOH; 1.0 mmol/L Na_2_EDTA; pH > 13) at 4 °C for 30 min. Electrophoresis was performed in this buffer for 20 min by applying 20 V and adjusting the current to 300 mA. After unwinding and electrophoresis, the broken DNA will migrate out of the nucleus. Under the microscope, the images of damaged cells are observed as a bright comet head with a dispersive tail, whereas healthy cells are observed as a round circle (Figure 2). Finally, the slide was gently washed twice in a buffer solution (0.4 mol/L Tris-HCl; pH = 7.5), fixed in methanol for 15 min, dried at room temperature, and stained with 0.005% ethidium bromide for 10 min. After dipping the samples in a strong alkaline electrophoresis solution (pH > 13), healthy cells were round with a fluorescent, smooth surface (as shown in Figure 2a). If the cells were damaged, the DNA structure exhibits a fractured phenomenon, and the speed of fragment movement to the anode side is faster than the large segments during electrophoresis. Then, the DNA forms the comet phenomenon and has a clear tail (as shown in Figure 2b).

For each slide, 200 nuclei, chosen at random, were analyzed. Comet assay images were obtained using a fluorescence microscope with an excitation filter of BP546/10 nm and a barrier filter of 590 nm [21,22].

A computerized image analysis system (CASP Version 1.2.3b1, San Francisco, CA, USA) was employed. From each sample, 200 cells were selected randomly for the assay, the number of damaged cells was counted, and statistical analysis of every tail DNA % and tail moment was conducted. The percentage of DNA in the tail (tail DNA %) and tail moment (TM) were used as the measures of DNA damage. Three slides were evaluated per treatment, and each treatment was repeated at least twice. From the repeated experiments, the averaged median values were calculated. Data were analyzed using the statistical software SPSS 18.0 (Chicago, IL, USA). The values were analyzed with the analysis of variance (ANOVA).

## 3. Results and Discussion

### 3.1. Results of DNA Damage Assay

#### 3.1.1. Tail DNA Percent Analysis

**Figure 3:**
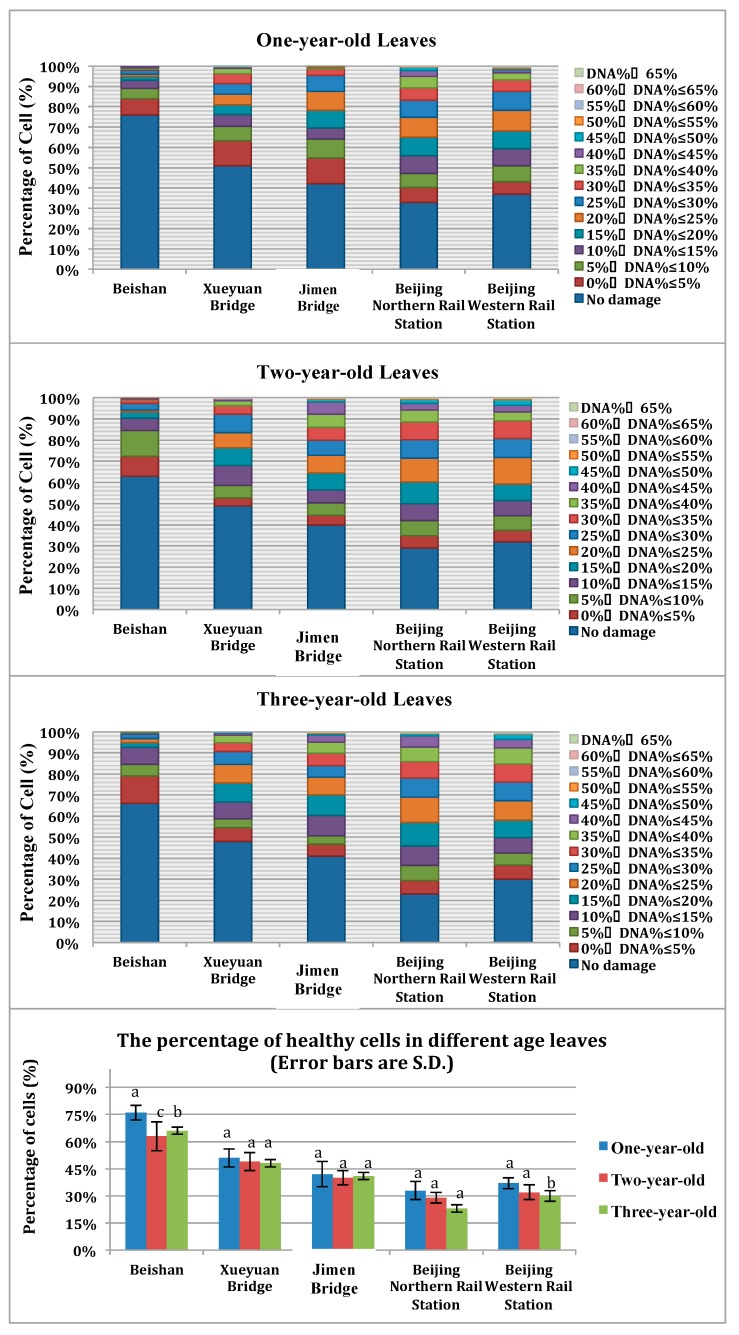
Cumulative DNA damage of cells in terms of the percentage of DNA in tail. The different letters above the error bars within the same sampling site means they differ significantly (*p* < 0.05).

According to the comet images, the number of cells with a tail out of every 200 cells from each sample was counted. Based on the visual scoring of the comet appearance, we identified different magnitudes of damage in the sampled population. According to the results, the maximum value for tail DNA percent (percentage of DNA in the tail) was 71.36 and the tail DNA percent was divided into 15 groups: 0%–5%, 5%–10%, 10%–15% ... 60%–65%, and 65%–100% (Figure 3).

The tail DNA percent reflects the proportion of damage in the assayed cells. In the control sample, no damage was observed in cells from Beishan, and the damage was much higher in samples taken along the road in the same growth year. The one-year-old leaves in the control sample showed 24% damaged cells, whereas the one-year-old leaves from the Beijing Northern Rail Station showed 67% damaged cells, and three-year-old leaves from the Beijing Northern Rail Station showed 77% damaged cells. The difference between control samples and roadside samples reache higher than 50% while, in other relative research 8, the most significant difference in degrees of DNA damage between roadside and non-roadside periwinkle samples is about 30%, and pothos is approximately 10%. In the present research, the four urban samples show a greater number of nuclei with medium damage and some nuclei with substantial damage (tail DNA percent >50%). Compared to control samples, the four urban samples have much higher damage because of the difference in growing environment, which is that the plants are growing on the side of busy roads. Therefore, the results indicated that the serious roadside pollution in Beijing has caused heavier DNA damage, and *Euonymus japonicus* leaves are sensitive to roadside pollution.

The occurrence of DNA damage in plants or animals exposed to environmental pollution may be relevant due to the fact that DNA strands may be broken due to the interaction with free radicals, organic and inorganic contaminants, heavy metals, etc., and the formation of adduct [23]. It is well known that environmental pollutants, such as metallic and organic xenobiotics, can induce the generation of reactive oxygen species (ROS), which could lead to oxidative damage to cellular macromolecules, including DNA, protein, and lipids [24]. As early as 1999, DNA strand breakage as a sensitive endpoint for DNA damage has been confirmed [25]. If the damaged DNA is not repaired properly, it will result in an adverse effect on DNA function.

In addition, we found that specimens collected from the same place showed a slight difference in DNA damage between different age groups (as show in Figure 3). Comparing the percentage of healthy cells at different age groups in these five sampling sites, it obviously showed that the one-year-old leaves had the highest percentage of healthy cells compared with the two-year-old and three-year-old leaves, although these differences are not statistically significant (*p* > 0.05, except for Beishan). We can intuitively find that the DNA damage level of one-year-old leaves are lower, which indicates the DNA damage in plants could accumulate over time. The statistical analysis results of the data showed that the differences are not significant, which may be due to the study years (1–3 years) not being long enough. The cumulative effect is not obvious enough, however, it is intuitively plausible that DNA damage in plants are associated with exposure time. Nevertheless, the quantitative relationship needs further study. Luqing’s research [24] about the DNA damage induced by three heavy metal ions (Cu^2+^, Pb^2+^, and Cd^2+^) of the *Charybdis japonica* tissues (gills, hepatopancreas, and hemocytes), which found that the levels of DNA single-strand breaks in all of the experimental groups increased significantly compared to the controls during the exposure time (0.5–15 days), indicates that the impact of effects with exposure time could accumulate. These findings for *Charybdis japonica* are in accordance with our research results that the genetic toxic effect could be accumulated with exposure time, both in plants and animals.

#### 3.1.2. Tail Moment Analysis

**Figure 4:**
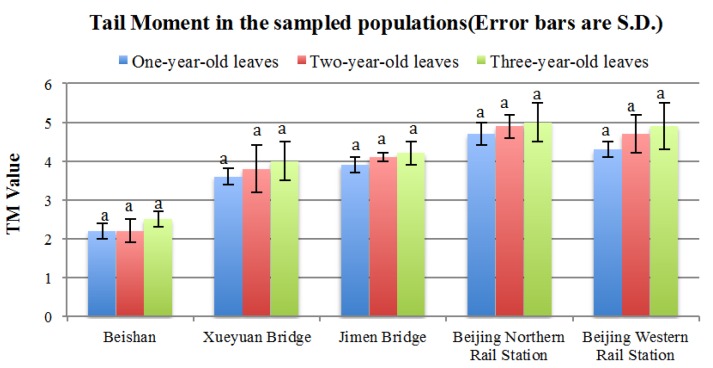
Tail moment in the sampled populations. The same letters above the error bars within the same sampling site means no significant difference (*p* > 0.05).

The same cells from each group were chosen for tail moment (TM) analysis. As TM is an integrated value of the tail DNA density multiplied by the migration distance, different magnitudes of DNA damage are depicted in Figure 4, in which the abscissa corresponds to the sampling points and the ordinate corresponds to TM values.

The TM values represent the degree of damage in each cell. The urban samples exhibited higher TM values compared to the negative control sample (Beishan, *p* < 0.01). Among the four test samples, there was some variation, although the differences were not significant (*p* > 0.05). In addition, the three groups of different ages from the same place showed an increased DNA damage value with increasing age, although there are no significant differences (*p* > 0.05), it is intuitively plausible that the degree of damage in cells is associated with exposure time. Nevertheless, the quantitative relationship needs further study, maybe by extending the number of study years.

Considering the damage to each cell, we can calculate each group’s DNA damage degree (DD). TM values were divided into five grades: Grade 0, when TM = 0; Grade 1, when TM ≤ 2; Grade 2, when TM ≤ 3; Grade 3, when TM ≤ 4; Grade 4, when TM > 4. Grade 0 represents the healthy cells.

The ratio of damaged cells in each grade was calculated as the damaged cell in each grade count divided by the total cell count (the total cell count is 200 for each sampling point). The damage degree (DD) of each sample was calculated using the formula below, and the results are shown in Table 1. According to the fluorescence intensity of the tail, the *DDunit* was divided into five intervals for each grade with the following values: *DDunit_(grade 0)_* = 0; *DDunit_(grade 1)_* = 100; *DDunit_(grade 2)_* = 200; *DDunit_(grade 3)_* = 300; and *DDunit_(grade 4)_* = 400.

$$DD=\overset{4}{\underset{i=0}{\sum }}DDunit_{\left(grade\;i\right)}\times \frac{damaged\;cell\;count_{\left(grade\;i\right)}}{total\;cell\;count}$$

Table 1 shows that the damage degree increases with growth time (exposure time) and that samples exposed to poor air quality are more damaged than control samples. The independent sample *T*-test indicated that roadside pollution has an accumulating influence on DNA damage over time but is not linearly dependent. For example, Beijing Northern Rail Station showed that the DNA damage degree of one-year-old leaves is 150, whereas for two-year-old and three-year-old samples, the values are 163.3 and 178.7.

### 3.2. Spatial Distribution Analysis of Dust Pollution

We calculated the Air Quality Index (AQI) for Beijing using the pollutant concentration data from the Beijing Environmental Statement 2012, and the following Equations:

$$IAQI_{p}=\frac{IAQI_{Hi}-IAQI_{Lo}}{BP_{Hi}-BP_{Lo}}\left(C_{P}-BP_{Lo}\right)+IAQI_{Lo}$$

$$AQI=max\left\{IAQI_{1},IAQI_{2},IAQI_{3},\cdots IAQI_{n}\right\}$$

in which IAQI_P_ is the index for pollutant p; C_P_ is the mass concentration of pollutant p; BP_Hi_ is the largest observed value of C_P_; BP_Lo_ is the lowest observed value of C_P_; IAQI_Hi_ is the individual AQI value corresponding to BP_Hi_; and IAQI_Lo_ is the individual AQI value corresponding to BP_Lo_.

The sampling points can be divided into two groups. The control group, Beishan, is in the Miyun district, and the other test sites are in the urban area group. In the Miyun district, IAQI_NO2_ is 50, but this value is 71 in the urban area. IAQI_PM10_ in the Miyun district is 68, but this value is 87 in the urban area. IAQI_PM2.5_ in the Miyun district is 61 and is 79 in the urban area. NO_2_ is the primary pollutant (IAQI > 50), whereas PM_10_ and PM_2.5_ are major pollutants (IAQI > 100). Some other research [26] has tested the compounds of the particulate matter (PM) in Beijing, including black carbon (BC), aluminum (Al), calcium (Ca), potassium (K), iron (Fe), sulfur (S), silicon (Si), titanium (Ti), and zinc (Zn). Hanbin [27] verified that exposure to traffic-related air pollution could cause DNA damage in human beings; the urinary 8-OHdG levels and the occurrence of DNA strand breaks in traffic conductors significantly exceeded those in indoor office workers in mixed models, and the particulate PAHs levels showed a positive association with urinary 1-OHPG in the regression model (β = 0.056, *p* = 0.01). In the present study, all monitoring points are in a very similar pollution environment, the largest difference between the urban samples and the control sample is IAQI_PM10_ and IAQI_PM2.5_, and many studies [26] showed that the particle composition in different locations are similar in this research area. Therefore, we use IAQI_PM10_ and IAQI_PM2.5_ to represent the pollution degrees between urban samples and the control sample.

The concentrations of PM_2.5_ and PM_10_ were measured using the TSI DustTrak. We distinguished between fine and poor air quality (hazy weather) according to the secondary concentration limit of the new ambient air quality standard (GB3095-2012), which is 150 μg/m^3^. We statistically analyzed each site’s PM_2.5_ and PM_10_ concentration in fine and poor air quality, and the mean values are shown in Table 2.

According to Table 2, we observed that the gap in pollution degree between different sampling sites is increased in hazy weather, which also indicates that along roadways in Beijing air pollution from vehicles is aggravated by haze. Regardless of AQI, the PM concentration of the control sample is much smaller than the urban samples (*p* < 0.01), approximately one third of the urban samples. Additionally, we identified that the PM_2.5_ concentration accounts for approximately 85% of the PM_10_ concentration for a fine AQI, and for a poor AQI it is 90% or more.

According to the proportion of fine and poor weather, fine weather accounts for two-fifths (40%) of the sampling period. Combined with the AQI calculation formula, the AQI values are listed in Table 3.

We found a statistically significant difference between the mean of the PM concentration for each of the urban samples and the control (*p* < 0.01), as well as for tail DNA percent (Figure 3) and TM damage (Figure 4). This indicates that high concentrations of roadside pollution resulted in a significant increase in DNA damage because both the tail DNA percent and the TM damage were related to the AQI. Furthermore, the DNA damage degree is consistent with the AQI. The values for the Beishan control sample are much lower than the urban samples, whereas the Beijing Northern Rail Station’s values are slightly higher than the urban samples. Tovalin [28] investigated 55 outdoor and indoor workers’ exposure to volatile organic compounds, PM_2.5_, and ozone from Mexico City and Puebla, finding that the DNA damage magnitude suffered by humans positively correlated with PM_2.5_ and ozone exposure. Outdoor and indoor workers with ≥60% of highly damaged cells (highly damaged workers) had significantly higher exposures to PM_2.5_, ozone, and some volatile organic compounds. These findings suggested there is a correlation between increased DNA strand breaks and personal exposure to PM_2.5_ for humans, and as our results show, the correlation is consistent in plants, as well.

**Figure 5:**
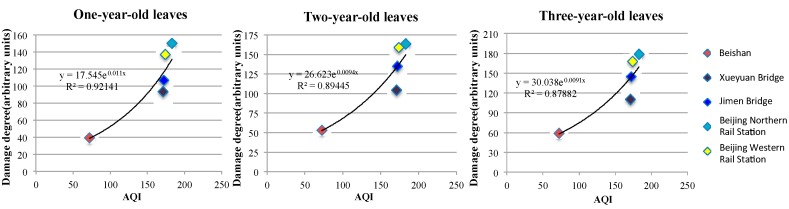
AQI and damage degree for each sample, comparing the DNA damage each year to the AQI (*p* < 0.01) of the five sample sites.

If we compare the DNA damage each year to the AQI (*p* < 0.01) of the five sample sites, ranging from 0.921 in the first year to 0.894 in the second year, and finally to 0.878 in the third year (Figure 5), the impact of the pollution conditions on plants decreases, and the correlation index increases with exposure time, indicating that DNA damage is very dependent on AQI. Moreover, from the three equations, we calculated the sensitivity coefficient δ=dyydxx and found that δ_year 1_ = 0.011*x*, δ_year 2_ = 0.009*x*, and δ_year 3_ = 0.009*x*. The sensitivity coefficients tell us that one-year-old leaves are more sensitive to pollution. From the K equations, K_1_ = 17.54, K_2_ = 26.62, and K_3_ = 30.03, we found that, as leaves age from one-year-old to three-years-old, there is an accumulation of effects.

## 4. Conclusions

The comet assay is a sensitive assay for detecting DNA single-strand breaks and alkali-labile damage in individual cells. Our results corroborate an alarmingly high level of DNA damage in *Euonymus japonicus* leaves from road plants in Beijing in 2013 and can be accounted for by roadside pollution, in which the most damaged cells can reach up to 77%, and the highest damage degree in roadside samples was 178.7 ± 2.2, whereas the control sample was 59 ± 1.6. In our research, according to the real-time AQI values, the DNA damage to *Euonymus japonicus* leaf cells was positively correlated with roadside pollution (*p* < 0.01). According to the sensitive coefficients (δ_year 1_ = 0.011*x*, δ_year 2_ = 0.009*x*, δ_year 3_ = 0.009*x*), it showed that the one-year-old leaves were more sensitive to pollution; and the K equation (K_1_ = 17.54, K_2_ = 26.62, K_3_ = 30.03) results proved that there is a cumulative effect as the leaves age. However, because of the limitation of study years (1–3 years), results in the accumulation effect of genetic damage over time is not significant; the results of our research can only intuitively show that there was a correlation between plant genetic damage and exposure time, but the quantitative indicators of the correlation degrees need further research.

Taking into account our observation of high levels of genetic damage in *Euonymus japonicus* due to mixed roadside environmental contamination that is affected by both traffic pollution and hazy weather, this damage likely manifests in humans as well, which is verified by many researchers. In our present study, the apparent genotoxicity endured by the plants along the road increased tail DNA percent and TM damage values when the leaves were collected near heavy traffic, although we do not know the exact composition of the pollutants causing the DNA damage in plants grown near congested roads in Beijing. In addition, the further study is required in order to determine the damage mechanisms of the mixed roadside air pollutants in plant cells.

However, our results might serve as support for the suitability and sensitivity of the comet assay for surveying plant DNA damage induced by environmental genotoxic agents.

## Figures and Tables

**Table 1:** Damage degree (DD) for each sample. The DD of each sample was calculated using the formula and the results as shown below. (Values expressed as the mean ± SD).

Leaf Ages	Beishan	Xueyuan Bridge	Jimen Bridge	Beijing Northern Rail Station	Beijing Western Rail Station
One year	39.5 ± 1.2	93.5 ± 1.6	106.8 ± 1.3	150 ± 1.4	137 ± 2.0
Two years	53.3 ± 1.8	104.3 ± 1.7	134.9 ± 2.2	163.3 ± 2.4	158.8 ± 1.8
Three years	59 ± 1.6	110.2 ± 2.0	144.6 ± 2.0	178.7 ± 2.2	167.8 ± 2.2

**Table 2:** PM concentration in the sampling vicinity. We statistically analyzed each site’s PM_2.5_ and PM_10_ concentration in fine and poor air quality, and the mean values are shown below. (Unit: μg/m^3^).

Sampling Sites	Fine AQI		Poor AQI	
	PM_2.5_	PM_10_	PM_2.5_	PM_10_
Beishan	21	24	87	93
Xueyuan Bridge	53	57	275	291
Jimen Bridge	52	55	276	293
Beijing Northern Rail Station	74	81	313	325
Beijing Western Rail Station	58	63	282	298

**Table 3:** AQI values of the samples. According to the proportion of fine and poor weather, fine weather accounts for two-fifths of the sampling period. Combined with the AQI calculation formula, the AQI values of each sample is shown below.

Sampling Sites	Beishan	Xueyuan Bridge	Jimen Bridge	Beijing Northern Rail Station	Beijing Western Rail Station
AQI	72	171	172	183	174
